# 1,3-Bis(chloro­meth­yl)benzene

**DOI:** 10.1107/S1600536813016383

**Published:** 2013-06-26

**Authors:** Marisa B. Sanders, David Leon, Eddy I. Ndichie, Benny C. Chan

**Affiliations:** a Department of Chemistry, The College of New Jersey, 2000 Pennington Rd, Ewing, NJ 08628, USA

## Abstract

The title compound, C_8_H_8_Cl_2_, used in the synthesis of many pharmaceutical inter­mediates, forms a three-dimensional network through chlorine–chlorine inter­actions in the solid-state that measure 3.513 (1) and 3.768 (3) Å.

## Related literature

For background information on the applications of halogenated xylenes, see: Ito & Tada (2009 ▶); Zordan & Brammer (2006 ▶). For related structures, see: Castaner *et al.* (1991 ▶). For halogen–halogen inter­actions, see: Hathwar *et al.* (2010 ▶). For additional information on how the space group of the structure was solved, see Spek (2009 ▶).
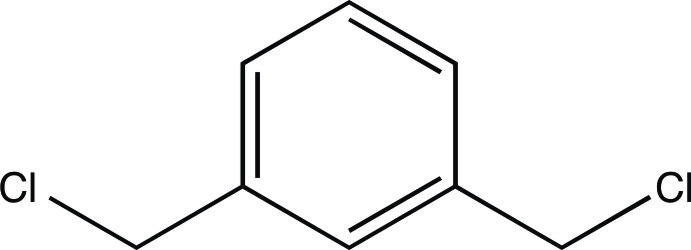


## Experimental

### 

#### Crystal data

C_8_H_8_Cl_2_
*M* *_r_* = 175.04
Orthorhombic, 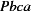

*a* = 8.5174 (5) Å
*b* = 12.3094 (7) Å
*c* = 15.2597 (9) Å
*V* = 1599.89 (16) Å^3^
*Z* = 8
Mo *K*α radiation
μ = 0.73 mm^−1^
*T* = 100 K
0.51 × 0.50 × 0.10 mm

#### Data collection

Bruker APEXII CCD diffractometer
Absorption correction: multi-scan (*SADABS*; Bruker, 2011 ▶) *T*_min_ = 0.665, *T*_max_ = 0.746
17126 measured reflections
1949 independent reflections
1782 reflections with *I* > 2σ(*I*)
*R*_int_ = 0.026

#### Refinement

*R*[*F* ^2^ > 2σ(*F* ^2^)] = 0.023
*wR*(*F* ^2^) = 0.064
*S* = 1.04
1949 reflections
91 parameters
H-atom parameters constrained
Δρ_max_ = 0.39 e Å^−3^
Δρ_min_ = −0.24 e Å^−3^

### 

Data collection: *APEX2* (Bruker, 2011 ▶); cell refinement: *SAINT* (Bruker, 2011 ▶); data reduction: *SAINT*; program(s) used to solve structure: *SHELXS97* (Sheldrick, 2008 ▶); program(s) used to refine structure: *SHELXL97* (Sheldrick, 2008 ▶); molecular graphics: *CrystalMaker* (CrystalMaker Software, 2009 ▶); software used to prepare material for publication: *enCIFer* (Allen *et al.* 2004 ▶).

## Supplementary Material

Crystal structure: contains datablock(s) I, global. DOI: 10.1107/S1600536813016383/mw2106sup1.cif

Structure factors: contains datablock(s) I. DOI: 10.1107/S1600536813016383/mw2106Isup2.hkl

Supplementary material file. DOI: 10.1107/S1600536813016383/mw2106Isup3.cml

Additional supplementary materials:  crystallographic information; 3D view; checkCIF report

## Figures and Tables

**Fig. 1.**
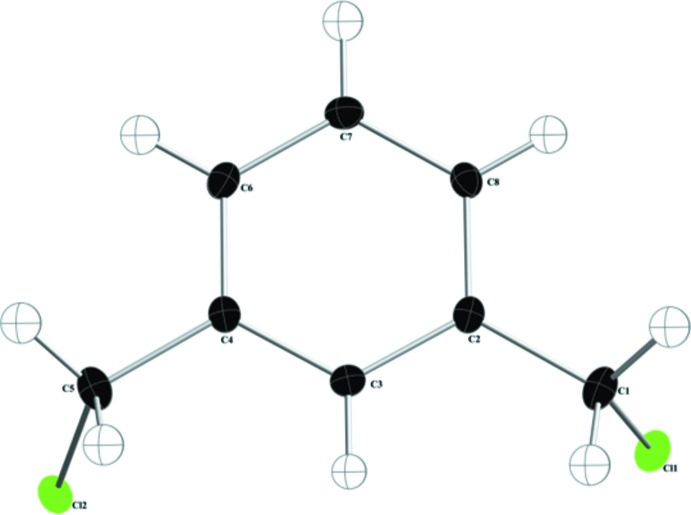
Thermal ellipsoid plot at 50% probability.

**Fig. 2.**
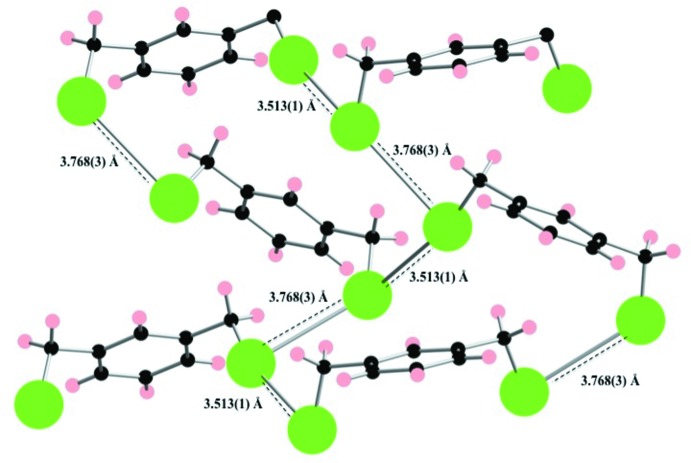
The title structure is stabilized by chlorine-chlorine interactions that measure 3.513 (1) Å and 3.768 (3) Å to form a three dimensional network. Carbon atoms are shown in black, hydrogen atoms in pink, and chlorine atoms in green.

## supplementary crystallographic information

### Comment

Halogenated xylenes, including the title compound, have a variety of applications in agrochemicals, drugs, and macromolecular materials (Ito and Tada, 2009). Most recently, these compounds have shown potential for their use in hard-coated film for optical devices (Ito and Tada, 2009). Compounds such as the title one that contain networks of halogen-halogen contacts also have a variety of applications in liquid crystals, topochemical reactions, conducting materials, and anion receptors (Zordan and Brammer, 2006). 1,3-Bis(chloromethyl)benzene is stabilized by chlorine-chlorine interactions of 3.513 (1) Å and 3.768 (3) Å. These contacts are within the normal range of chlorine-chlorine interactions, which are typically 3.546 Å to 3.813 Å (Hathwar *et al.*, 2010).

### Experimental

Approximately 100 mg of the title compound was dissolved in 2 ml of hexanes. The solution was evaporated slowly over one week to produce large, clear, block crystals.

### Refinement

The structure was solved using direct methods (Bruker, 2011).

### Crystal data

**Table:**

C_8_H_8_Cl_2_	*D*_x_ = 1.453 Mg m^−^^3^*D*_m_ = 1.453 Mg m^−^^3^*D*_m_ measured by not measured
*M**_r_* = 175.04	Mo *K*α radiation, λ = 0.71073 Å
Orthorhombic, *P**b**c**a*	Cell parameters from 120 reflections
*a* = 8.5174 (5) Å	θ = 2.7–28.2°
*b* = 12.3094 (7) Å	µ = 0.73 mm^−^^1^
*c* = 15.2597 (9) Å	*T* = 100 K
*V* = 1599.89 (16) Å^3^	Thick plate, colourless
*Z* = 8	0.51 × 0.50 × 0.10 mm
*F*(000) = 720	

### Data collection

**Table:**

Bruker APEXII CCD diffractometer	1949 independent reflections
Radiation source: fine-focus sealed tube	1782 reflections with *I* > 2σ(*I*)
Graphite monochromator	*R*_int_ = 0.026
Detector resolution: 8.3333 pixels mm^-1^	θ_max_ = 28.6°, θ_min_ = 2.7°
ω and φ scans	*h* = −11→11
Absorption correction: multi-scan (*SADABS*; Bruker, 2011)	*k* = −15→15
*T*_min_ = 0.665, *T*_max_ = 0.746	*l* = −20→20
17126 measured reflections	

### Refinement

**Table:**

Refinement on *F*^2^	Primary atom site location: structure-invariant direct methods
Least-squares matrix: full	Secondary atom site location: difference Fourier map
*R*[*F*^2^ > 2σ(*F*^2^)] = 0.023	Hydrogen site location: inferred from neighbouring sites
*wR*(*F*^2^) = 0.064	H-atom parameters constrained
*S* = 1.04	*w* = 1/[σ^2^(*F*_o_^2^) + (0.0356*P*)^2^ + 0.6168*P*] where *P* = (*F*_o_^2^ + 2*F*_c_^2^)/3
1949 reflections	(Δ/σ)_max_ = 0.001
91 parameters	Δρ_max_ = 0.39 e Å^−^^3^
0 restraints	Δρ_min_ = −0.24 e Å^−^^3^

### Special details

**Table:**

Geometry. All e.s.d.'s (except the e.s.d. in the dihedral angle between two l.s. planes) are estimated using the full covariance matrix. The cell e.s.d.'s are taken into account individually in the estimation of e.s.d.'s in distances, angles and torsion angles; correlations between e.s.d.'s in cell parameters are only used when they are defined by crystal symmetry. An approximate (isotropic) treatment of cell e.s.d.'s is used for estimating e.s.d.'s involving l.s. planes.
Refinement. Refinement of *F*^2^ against ALL reflections. The weighted *R*-factor *wR* and goodness of fit *S* are based on *F*^2^, conventional *R*-factors *R* are based on *F*, with *F* set to zero for negative *F*^2^. The threshold expression of *F*^2^ > σ(*F*^2^) is used only for calculating *R*-factors(gt) *etc*. and is not relevant to the choice of reflections for refinement. *R*-factors based on *F*^2^ are statistically about twice as large as those based on *F*, and *R*- factors based on ALL data will be even larger.

### Fractional atomic coordinates and isotropic or equivalent isotropic displacement parameters (Å^2^)

**Table:**

	*x*	*y*	*z*	*U*_iso_*/*U*_eq_	
Cl1	0.61622 (3)	0.35365 (2)	1.030229 (18)	0.02078 (9)	
Cl2	0.83843 (3)	0.32450 (2)	0.660472 (18)	0.02164 (10)	
C1	0.81471 (14)	0.40672 (11)	1.02366 (8)	0.0210 (3)	
H1A	0.8343	0.4563	1.0736	0.025*	
H1B	0.8909	0.3462	1.0272	0.025*	
C2	0.83655 (13)	0.46689 (10)	0.93903 (7)	0.0159 (2)	
C3	0.90389 (13)	0.41528 (9)	0.86690 (8)	0.0161 (2)	
H3	0.9394	0.3424	0.8722	0.019*	
C4	0.91971 (13)	0.46956 (9)	0.78693 (7)	0.0165 (2)	
C5	0.98635 (14)	0.41163 (11)	0.70880 (8)	0.0218 (3)	
H5A	1.0779	0.3675	0.7269	0.026*	
H5B	1.0224	0.4654	0.6650	0.026*	
C6	0.78830 (13)	0.57490 (10)	0.93140 (8)	0.0182 (2)	
H6	0.7434	0.6110	0.9804	0.022*	
C7	0.80570 (15)	0.62972 (10)	0.85252 (8)	0.0209 (2)	
H7	0.7736	0.7034	0.8478	0.025*	
C8	0.87016 (14)	0.57693 (10)	0.78021 (8)	0.0196 (2)	
H8	0.8803	0.6144	0.7261	0.024*	

### Atomic displacement parameters (Å^2^)

**Table:**

	*U*^11^	*U*^22^	*U*^33^	*U*^12^	*U*^13^	*U*^23^
Cl1	0.01567 (15)	0.02646 (17)	0.02020 (16)	−0.00530 (11)	0.00008 (10)	0.00373 (11)
Cl2	0.02070 (16)	0.02609 (17)	0.01814 (15)	−0.00366 (11)	0.00164 (10)	−0.00641 (11)
C1	0.0148 (5)	0.0300 (7)	0.0181 (6)	−0.0067 (5)	−0.0028 (4)	0.0046 (5)
C2	0.0113 (5)	0.0219 (6)	0.0145 (5)	−0.0039 (4)	−0.0025 (4)	0.0009 (4)
C3	0.0121 (5)	0.0165 (5)	0.0196 (6)	−0.0015 (4)	−0.0019 (4)	0.0000 (4)
C4	0.0117 (5)	0.0216 (6)	0.0162 (5)	−0.0028 (4)	−0.0004 (4)	−0.0022 (4)
C5	0.0150 (5)	0.0298 (7)	0.0206 (6)	−0.0035 (5)	0.0013 (4)	−0.0065 (5)
C6	0.0158 (5)	0.0216 (6)	0.0173 (5)	−0.0008 (4)	0.0018 (4)	−0.0041 (4)
C7	0.0209 (6)	0.0166 (6)	0.0250 (6)	0.0013 (4)	0.0000 (5)	0.0012 (5)
C8	0.0192 (5)	0.0227 (6)	0.0171 (5)	−0.0017 (5)	0.0000 (4)	0.0039 (5)

### Geometric parameters (Å, º)

**Table:**

Cl1—C1	1.8152 (12)	C4—C8	1.3912 (17)
Cl2—C5	1.8115 (12)	C4—C5	1.5007 (16)
C1—C2	1.5004 (16)	C5—H5A	0.9900
C1—H1A	0.9900	C5—H5B	0.9900
C1—H1B	0.9900	C6—C7	1.3879 (17)
C2—C3	1.3944 (16)	C6—H6	0.9500
C2—C6	1.3964 (17)	C7—C8	1.3933 (17)
C3—C4	1.3978 (16)	C7—H7	0.9500
C3—H3	0.9500	C8—H8	0.9500
			
C2—C1—Cl1	109.92 (8)	C4—C5—Cl2	109.97 (8)
C2—C1—H1A	109.7	C4—C5—H5A	109.7
Cl1—C1—H1A	109.7	Cl2—C5—H5A	109.7
C2—C1—H1B	109.7	C4—C5—H5B	109.7
Cl1—C1—H1B	109.7	Cl2—C5—H5B	109.7
H1A—C1—H1B	108.2	H5A—C5—H5B	108.2
C3—C2—C6	119.28 (11)	C7—C6—C2	120.25 (11)
C3—C2—C1	120.37 (11)	C7—C6—H6	119.9
C6—C2—C1	120.35 (11)	C2—C6—H6	119.9
C2—C3—C4	120.73 (11)	C6—C7—C8	120.14 (11)
C2—C3—H3	119.6	C6—C7—H7	119.9
C4—C3—H3	119.6	C8—C7—H7	119.9
C8—C4—C3	119.29 (11)	C4—C8—C7	120.28 (11)
C8—C4—C5	120.50 (11)	C4—C8—H8	119.9
C3—C4—C5	120.19 (11)	C7—C8—H8	119.9
